# Hypoglycemia in Patients with Diabetes and Renal Disease

**DOI:** 10.3390/jcm4050948

**Published:** 2015-05-13

**Authors:** Mazen Alsahli, John E. Gerich

**Affiliations:** 1 Department of Medicine, Southlake Health Center and University of Toronto Faculty of Medicine, 531 Davis Dr, Newmarket, Ontario L3Y 6P5, Canada; E-Mail: malsahli@utoronto.ca; 2 Department of Medicine, University of Rochester School of Medicine, 601 Elmwood Ave, Rochester, NY 14642, USA

**Keywords:** chronic kidney disease, diabetes, diabetic nephropathy, hypoglycemia, renal

## Abstract

This article summarizes our current knowledge of the epidemiology, pathogenesis, and morbidity of hypoglycemia in patients with diabetic kidney disease and reviews therapeutic limitations in this situation.

## 1. Introduction

Hypoglycemia is a common occurrence in people with diabetes and most frequently it is the result of pharmacologic intervention. Avoidance of and fear of hypoglycemia are often the major impediment for achieving optimal glycemic control [1]. Moreover, hypoglycemia is associated with significant morbidity and mortality [2,3,4,5,6]. 

Chronic kidney disease (CKD) is an independent risk factor for hypoglycemia, and augments the risk already present in people with diabetes [7,8,9]. In addition, CKD imposes restrictions on antidiabetic therapeutic options and increases the risk of cardiovascular disease and death [7,10,11,12,13]. This review represents an update and expansion of a recent publication of ours on this subject with more detailed discussion on therapeutic options limitations facing care providers in this common clinical situation [14]. PubMed and MEDLINE were searched for literature published in English from January 1989 to January 2015 for diabetes mellitus, hypoglycemia, chronic kidney disease, diabetic nephropathy, diabetic kidney disease, and chronic renal insufficiency. 

## 2. Definition and Classification of Hypoglycemia in Diabetes

The American Diabetes Association and Endocrine Society workgroup on hypoglycemia defined iatrogenic hypoglycemia in patients with diabetes as all episodes of an abnormally low plasma glucose concentration that expose the patient to potential harm [15]. No single threshold value was assigned to define hypoglycemia since this value may differ among patients. An alert value of <70 mg/dL (<3.8 mmol/L), however, was chosen to draw the attention of patients and caregivers and also for use as a cut-off value in the classification of hypoglycemia in diabetes as outlined in Table 1 [15].

**Table 1:** Hypoglycemia categories as defined by the American Diabetes Association and the Endocrine Society [15].

Category	Definition
Documented symptomatic	An event during which typical symptoms of hypoglycemia are associated by a measured plasma glucose concentration ≤70 mg/dL ^a^
Severe	An event requiring assistance of another person to administer carbohydrate, glucagon, or other resuscitative actions ^b^
Asymptomatic	An event not accompanied by typical symptoms of hypoglycemia but with a measured plasma glucose concentration ≤70 mg/dL ^a^
Probable symptomatic	An event during which symptoms of hypoglycemia are not accompanied by a plasma glucose measurement but that was presumably caused by a plasma glucose concentration ≤70 mg/dL ^a^
Pseudo-hypoglycemia	An event during which the person with diabetes reports any of the typical symptoms of hypoglycemia with a measured plasma glucose concentration >70 mg/dL ^a^ but approaching that level

^a^ 70 mg/dL equals 3.8 mmol/L; ^b^ If plasma glucose measurements are not available during such an event; the neurological recovery attributable to the restoration of plasma glucose to normal is considered sufficient evidence that the event was induced by hypoglycemia.

## 3. Definition and Classification of CKD

The Kidney Disease Improving Global Outcomes (KDIGO) has defined CKD as abnormalities of kidney structure or function, present for >3 months, with implications for health [16]. The group classified CKD based on cause, estimated glomerular filtration rate (eGFR), and albuminuria. Diabetic kidney disease (DKD) refers to CKD caused by diabetes. DKD is usually a presumptive diagnosis detected clinically by screening for increased albuminuria and decreased eGFR. Since there may be other causes of CKD in patients with diabetes (e.g., hypertension, pyelonephritis), kidney biopsies may sometimes be needed to establish a definitive diagnosis [16]. 

Increased albuminuria is usually detected through abnormal reagent strip test for total protein or a random urine albumin/creatinine ratio (ACR) assessment. Although the appearance of increased albuminuria is usually the earliest finding of DKD, the severity of albuminuria does not necessarily predict DKD progression in patients with either type 1 or type 2 diabetes [17,18,19]. The normal ACR in young adults is <10 mg/g (<1 mg/mmol) [16]. Abnormal results should be confirmed by repeat testing at least twice over a 6 month period because of frequent false positives [20]. An elevated random ACR can be confirmed by urine albumin excretion rate in a timed urine collection, as necessary. Albuminuria categories in CKD according to KDIGO are summarized in Table 2.

**Table 2:** Albuminuria categories in chronic kidney disease (CKD) based on KDIGO ^a^ classification [16]. Adapted by permission from Macmillan Publishers Ltd.: Kidney International. KDIGO.

Albumin Excretion Rate (mg/24 h)	Albumin Creatinine Ratio (mg/mmol or mg/g)	Category (Description)
<30	<3 mg/mmol (<30 mg/g)	A1 (Normal to mildly increased)
30–300	3–30 mg/mmol (30–300 mg/g)	A2 (Moderately increased)
>300	>30 mg/mmol (>300 mg/g)	A3 (Severely increased)

^a^ KDIGO = Kidney Disease Improving Global Outcomes.

Estimating GFR from serum creatinine is appropriate for staging and tracking the progression of CKD in most clinical situations including in patients with DKD. The 2009 Chronic Kidney Disease Epidemiology Collaboration (CKD-EPI) formula and its modifications, which have been adopted by many clinical laboratories, were found more accurate than the Modification of Diet in Renal Disease (MDRD) Study equation and its modifications [16,21,22]. Using 2009 CKD-EPI equation is thus recommended by the KDIGO over the MDRD study equation for estimating GFR [16]. GFR categories according to KDIGO are outlined in Table 3.

**Table 3:** GFR categories in CKD based on KDIGO ^a^ classification [16]. Adapted by permission from Macmillan Publishers Ltd: Kidney International. KDIGO. Summary of recommendation statements. Kidney Int. 2013; 3(1):1–150, © 2013.

GFR (mL/min/1.73 m^2^)	Category (Description)
≥90	G1 * (Normal or high)
60–89	G2 * (Mildly decreased)
45–59	G3a (Mildly to moderately decreased)
30–44	G3b (Moderately to severely decreased)
15–29	G4 (Severely decreased)
<15	G5 (Kidney failure)

^a^ KDIGO = Kidney Disease Improving Global Outcomes; * Glomerular filtration rate (GFR) categories G1 and G2 do not constitute CKD in the absence of evidence of kidney damage.

## 4. Epidemiology

The U.S. National Health and Nutrition Examination Survey (NHANES) of 2011–2012 found that about 19% of participants with diabetes (type 1 or 2) had an estimated glomerular filtration rate (eGFR) of <60 mL/min/1.73 m^2^ [23]. The prevalence of kidney disease, characterized by either reduced kidney function (eGFR of <60 mL/min/1.73 m^2^) or albuminuria (ACR ≥3 mg/mmol (≥30 mg/g)), was nearly 50% among patients with diabetes [23]. The prevalence appears to be similar in several other countries. In the United Kingdom, one study showed that patients with diabetes (type 1 or 2) were four times more likely to have clinically significant CKD (defined as an eGFR <60 mL/min/1.73 m^2^) than those without diabetes. Nearly one-third of people with diabetes (31%) had eGFR <60 mL/min/1.73 m^2^ compared with only 6.9% of the general population [24]. In 2011, diabetes was the primary cause of new cases of end stage renal disease (ESRD) in approximately 60% of patients in Malaysia, Mexico, and Singapore; and in more than 40% of patients in the Republic of Korea, Hong Kong, the Philippines, Japan, the United States, and New Zealand [25]. 

The exact incidence and prevalence of hypoglycemia in patients with diabetes and/or renal disease are difficult to define because mild to moderate hypoglycemia may go unnoticed or unreported. Overall, hypoglycemia unawareness can be found in 25% of patients with diabetes [26]. The complete detection of chemical hypoglycemia would require continuous blood glucose measurements over prolonged periods. Studies using this approach have generally found that the frequency and duration of hypoglycemia, especially nocturnal hypoglycemia, are greater than what was previously thought [27,28]. More reliable data are available from studies reporting severe hypoglycemia that is associated with loss of consciousness or requiring external assistance [15]. In general, the frequency of hypoglycemia is lower in people with T2DM than in those with type 1 diabetes [29,30,31,32,33,34]. For example, the UK Hypoglycemia Study Group reported severe hypoglycemia rates in patients with type 2 diabetes on insulin >2 years (10 episodes per 100 patient-year) to be far less than in patients with type 1 diabetes (<5 years disease duration, 110 episodes per 100 patient-year; >15 years disease duration, 320 episodes per 100 patient-year) [33]. 

**Figure 1:**
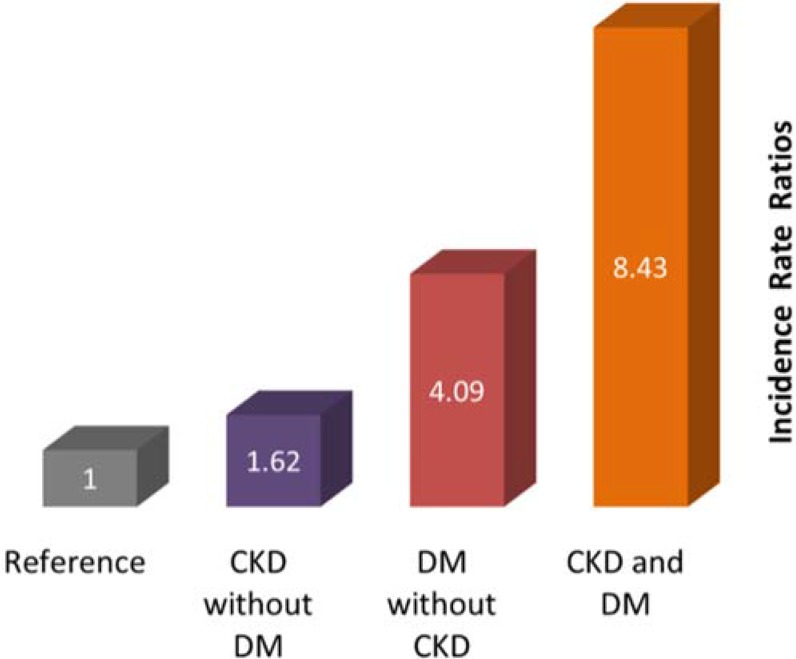
Risk for hypoglycemia of varying severity and expressed as an adjusted incidence rate ratio in patients classified by presence or absence of CKD and diabetes. Reference group are patients without CKD or diabetes. Rates adjusted for race, gender, age, Charlson comorbidity index, cancer, diabetes, and cardiovascular disease (all rate ratios *p <* 0.0001) [7].

Renal hypoglycemia (hypoglycemia associated with CKD without any other obvious cause) is known to occur spontaneously in non-diabetic individuals with an incidence of 1%–3% [35,36]. The presence of diabetes adds another layer of complexity. For example, Moen *et al.* found that the incidence of hypoglycemia is increased in the presence of either diabetes (type 1 or 2) or CKD, with the risk most pronounced in the presence of both conditions (Figure 1). Among patients with diabetes, the rate was 10.7 *versus* 5.3 per 100 patient-months and among patients without diabetes was 3.46 *versus* 2.23 per 100 patient-months, for CKD *versus* no CKD, respectively [7]. In a study by Muhlhauser *et al.*, type 1 diabetes patients with impaired kidney function had a fivefold higher incidence of severe hypoglycemia than type 1 diabetes patients with normal serum creatinine [37].

Among people with T2DM the frequency of hypoglycemia will vary by treatment modality. In general the frequency of hypoglycemia is greatest with insulin and insulin secretagogues that are excreted primarily by the kidney and/or have active metabolites that may accumulate in patients with impaired renal function such as glibenclamide (glyburide) [2,34,38]. Prandial insulin (short-acting insulin administered before meals to limit postprandial hyperglycemia) is associated with a greater frequency of hypoglycemia than long-acting basal insulin [39]. Metformin, thiazolidinediones, dipeptidyl peptidase-4 inhibitors, glucagon-like peptide 1 (GLP-1) mimetics and sodium glucose cotransporter-2 (SGLT2) inhibitors do not increase the risk of hypoglycemia when used without sulfonylureas or insulin [30,34,40].

## 5. Pathogenesis

### 5.1. Hypoglycemia Counterregulation

Normally plasma glucose levels are maintained within a relatively narrow range (between 70 and 140 mg/dL (3.8 and 7.8 mmol/L)) despite considerable variations in frequency and magnitude of carbohydrate intake and in energy expenditure. The maintenance of the stability of plasma glucose is due to the glucose counterregulatory system. By the time plasma glucose levels reach 70 mg/dL (3.8 mmol/L), secretion of counterregulatory hormones is stimulated—the key ones being glucagon which increases hepatic glucose production and catecholamines which mainly increase renal glucose release but also reduce muscle glucose uptake [1]. 

Within a few years of diabetes onset, people with type 1 diabetes develop impaired counter-regulatory hormone responses, which are manifested first by decreased or absent glucagon responses to hypoglycemia [1]. This is followed by decreased catecholamine responses and later (and variably) by decreased growth hormone and cortisol responses. The mechanism of the loss of glucagon response is poorly understood but recent evidence suggests that it could be related to increased activity of ATP-regulated potassium channels in glucagon-producing alpha cells [41]. The pathogenesis for impaired catecholamines and other hormones responses is also not entirely clear but may be a result of recurrent hypoglycemia that: (a) impairs glucose sensing in the ventromedial hypothalamus (a brain region that plays a major role in controlling the counterregulatory responses to hypoglycemia); and (b) leads to cellular adaptation which results in hypoglycemia unawareness and reduced adrenomedullary response to subsequent hypoglycemia [42,43]. 

Defective glucose counterregulation plays a major role in the susceptibility to severe hypoglycemia in patients with T1DM. By contrast, people with T2DM experience more modest impairment in glucose counterregulation [44]. While the counterregulatory responses to hypoglycemia have not been evaluated in people with CKD, there are various factors that would predispose those individuals to impaired counterregulation; for example, impairment of glucose release into the circulation by both the liver and kidney [45]. 

### 5.2. Renal Insufficiency as a Risk Factor for Hypoglycemia

Presence of CKD adds risk factors for hypoglycemia to these already existing in patients with diabetes. Some of the additional factors are altered drug metabolism, drug-drug interactions (e.g., angiotensin-converting enzyme inhibitors), albuminuria, autonomic neuropathy, anorexia, malnutrition, infections, problems linked to dialysis, associated cardiac and hepatic disease, and impaired renal glucose release [46,47].

In healthy people, both the liver (via glucagon) and kidney (via catecholamines) equally contribute to the increase in glucose release into the circulation during counterregulation of hypoglycemia; this is largely achieved by gluconeogenesis [1,45,48]. People with moderate to severe CKD have reduced renal mass, and therefore, a reduced capacity for renal glucose release. Moreover, these individuals could be malnourished and/or have muscle wasting, which decreases their hepatic glycogen stores and reduces the availability of gluconeogenic substrates [49]. Finally, acidosis would limit the ability of the liver to compensate via hepatorenal reciprocity (reciprocal changes in hepatic and renal glucose release to maintain normoglycemia) [45]. 

A decrease in renal clearance of insulin is evident when GFR falls below 15–20 mL/min/1.73 m^2^ [50]. At this point, a decline in hepatic insulin metabolism is also noted and is thought to be due to uremic toxins effects on the liver [50]. Management of CKD with dialysis reduces insulin resistance and increases insulin degradation and this includes an improvement in hepatic insulin metabolism [50,51]. Additionally, glucose is the most commonly used osmotic agent in peritoneal dialysis and glucose containing dialysis solutions can in many cause alternating hyperglycemia and hypoglycemia unless antidiabetes regimen and dialysis schedule is carefully managed [52]. 

Management of CKD consequences may also affect diabetes and alter insulin requirements. Examples of which include; increased insulin-induced glucose utilization following correction of anemia by erythropoietin [53,54], and improved insulin sensitivity following intravenous administration of calcitriol [55,56,57]. 

Over a 10-year follow-up period, Yun *et al.* demonstrated that the presence of baseline macroalbuminuria (defined as urinary albumin excretion ≥300 mg/day) was an independent risk factor for future development of severe hypoglycemia in T2DM patients with apparently normal or only minimally decreased renal function (e.g., GFR >60 mL/min/1.73 m^2^) irrespective of whether or not they were receiving insulin [9]. The exact underlying pathogenic mechanism for this is unclear. 

## 6. Hypoglycemia Morbidity and Mortality

Both hypoglycemia and CKD are associated with increased morbidity and mortality, particularly from cardiovascular disease [3,4,12,58,59,60]. Renal disease is associated with classic major cardiovascular risk factors, including hypertension, hyperlipidemia, and diabetes. Whether hypoglycemia per se is an additional risk factor or only a marker of cardiovascular frailty is currently a matter of debate [61,62]. There are theoretical, experimental and clinical considerations that suggest a causal effect, such as effects of hypoglycemia on oxidative stress, endothelial dysfunction, ST-segment prolongation and precipitation of arrhythmias via activation of the sympathetic nervous system [59,63,64].

## 7. Therapeutic Considerations

Prescribing protocols change in patients with CKD mostly to account for predicted pharmacokinetic changes. Recognizing these changes and applying principles of good prescribing is needed to reduce risk of hypoglycemia in patients on insulin or insulin secretagogues. Guidelines for use of antidiabetic agents vary among medical communities [65,66,67,68,69,70]. Information about dosing adjustments in patients with CKD and diabetes is summarized in Table 4.

**Table 4:** Recommended dosing adjustments of antidiabetic drugs in patients with diabetes and CKD.

Class and Agents	References	Therapeutic Considerations
Biguanides	[65,66,67,68,69,71,72,73]	
Metformin		Review use/reduce dose if eGFR < 45–60 mL/min/1.73 m^2^ Avoid if eGFR < 30 mL/min/1.73 m^2^ FDA is more restrictive indicating that metformin is contraindicated if serum creatinine ≥1.5 mg/dL (133 μmol/L) in males or ≥1.4 mg/dL (124 μmol/L) in females
Sulfonylureas	[65,69,70,74]	
Glyburide (glibenclamide)		Not recommended if eGFR <60 mL/min/1.73 m^2^
Gliclazide		Reduce dose if eGFR <30 mL/min/1.73 m^2^ Not recommended if eGFR <15 mL/min/1.73 m^2^
Glimepiride		Reduce dose if eGFR <30 mL/min/1.73 m^2^ Start at 1 mg daily or consider alternative agent if eGFR < 15 mL/min/1.73 m^2^
Glipizide		Can be used in all stages of CKD with caution. May need dose reduction
Meglitinides	[65,70,75,76]	
Repaglinide and Nateglinide		Can be used in all stages of CKD with caution. May need dose reduction if eGFR <30 mL/min/1.73 m^2^
DPP-4 inhibitors	[65,69,70]	
Sitagliptin		Reduce dose to 50 mg daily if eGFR 30–50 mL/min/1.73 m^2^ and to 25 mg daily if eGFR <30 mL/min/1.73 m^2^
Saxagliptin		Reduce dose to 2.5 mg daily if eGFR <50 mL/min/1.73 m^2^ Administer postdialysis in hemodialysis requiring patients
Linagliptin		No restrictions
Vildagliptin		Reduce dose to 50 mg daily when eGFR <50 mL/min/1.73 m^2^
Thiazolidinediones	[65,69,70]	
Rosiglitazone and Pioglitazone		No dose adjustment required
α-glucosidase inhibitors	[65,70,74,77,78]	
Acarbose and Miglitol		Not recommended if eGFR <25 mL/min/1.73 m^2^ or serum creatinine >2 mg/dL
Voglibose		Not well studied but is minimally absorbed and dose reduction unlikely needed
GLP-1 analogs	[70,71,79,80,81,82,83,84,85,86]	
Exenatide		Not recommended if eGFR <30 mL/min/1.73 m^2^
Liraglutide		Not recommended if eGFR <50 mL/min/1.73 m^2^
Albiglutide and Dulaglutide		Experience is limited. No dose adjustment required per FDA approval but the European Medicines Agency recommended avoiding their use in patients with GFR <30 mL/min/1.73 m^2^)
SGLT2 inhibitors	[72,87,88,89,90]	
Dapagliflozin		Not recommended if eGFR <60 mL/min/1.73 m^2^
Canagliflozin		Reduce dose to 100 mg once daily if eGFR 45–60 mL/min/1.73 m^2^ Not recommended if eGFR <45 mL/min/1.73 m^2^
Empagliflozin		Reduced dose to 10 mg once daily if eGFR 45–60 mL/min/1.73 m^2^ Not recommended if eGFR <45 mL/min/1.73 m^2^
Insulin	[42,52,70]	
Insulin		Use with caution. Dose reduction usually necessary if eGFR <30 mL/min/1.73 m^2^

GLP-1 = Glucagon-like peptide-1; DPP-4 = Dipeptidyl peptidase 4; SGLT2 = Sodium-glucose co-transporter 2.

- Metformin. The only route of elimination of metformin is via the kidneys. Consequently it may accumulate in people with impaired renal function. Most guidelines recommend reviewing or reducing metformin dose when eGFR is <60 mL/min/1.73 m^2^ (example, Canadian Diabetes Association and Swiss Society for Endocrinology and Diabetology [65,69]) or <45 mL/min/1.73 m^2^ (example, British National Institute for Health and Clinical Excellence, Australian Diabetes Society, and Japanese Society of Nephrology [66,67,68]) and avoiding its use altogether when eGFR is <30 mL/min/1.73 m^2^. The US Food and Drug Administration has stricter prescribing information limiting metformin use to men and women with serum creatinine <1.5 mg/dL (133 umol/L) and <1.4 mg/dL (124 umol/L), respectively [71]. On the other hand, a consensus statement of the American Diabetes Association mentions that metformin appears safe unless eGFR becomes <30 mL/min/1.73 m^2^ based on a review by Lipska *et al.* [72,73].
- Sulfonylureas. Hypoglycemia risk is increased as a consequence of accumulation of the sulfonylurea and/or its active metabolites and their long duration of action [74]. Glibenclamide (glyburide) and its two active metabolites (M1 and M2) are cleared by the kidneys. Its use is not recommended for people with eGFRs < 60 mL/min/1.73 m^2^ [65,69,70]. Glimepiride and gliclazide can be used with caution in people with mild-moderate renal insufficiency, and dose reduction is usually necessary especially when eGFR is <30 mL/min/1.73 m^2^; however, it is recommended to consider alternative agents in people with moderate-severe renal insufficiency specifically when eGFR is <15 mL/min/1.73 m^2^ [65,70]. The metabolism of glipizide occurs mainly in the liver and its primary metabolites are either inactive or with very weak hypoglycemic effect that are excreted in the urine; therefore, glipizide is the preferred sulfonylurea, but usually at a reduced-dose, in people with CKD [70,74].
- Meglitinides. Repaglinide can accumulate in patients with advanced renal dysfunction (eGFR <30 mL/min/1.73 m^2^) without significant increase in hypoglycemia [75]. A metabolite of nateglinide, that has modest hypoglycemic effect, accumulates in patients with CKD [76]. Both drugs may be used in CKD patients even in those with end-stage renal disease but with caution and at a reduced dose with careful upward titration [65,70].
- Dipeptidyl peptidase 4 (DPP-4) inhibitors. Sitagliptin, vildagliptin, and saxagliptin require reduction in dose once eGFRs are <50 mL/min/1.73 m^2^ because accumulation may in theory increase side effects. However, linagliptin does not require dose adjustment since its renal excretion is minimal. All these agents may be used in patients with severe renal impairment [65,69,70].
- Thiazolidinediones. Pioglitazone and rosiglitazone require no dose adjustment in renal disease and are not associated with a risk of hypoglycemia when used as monotherapy [65,69,70].
- Alpha glucosidase inhibitors. Acarbose and miglitol are not generally recommended for people with CKD due to potential accumulation and lack of safety information. Serum levels of acarbose and its metabolites are increased in CKD patients despite its minimal intestinal absorption [74,77]. Miglitol undergoes kidney excretion after substantial intestinal absorption (>50%) [74,77]. Data are lacking on the significance of accumulation of these drugs on hypoglycemia risk. Both medications are not recommended when the eGFR is <25 mL/min/1.73 m^2^ [65,70,74,77]. Voglibose is poorly absorbed after clinically relevant oral dose suggesting that no dose adjustment is required. However, studies in patients with renal insufficiency are not available [78].
- Glucagon-like peptide-1 (GLP-1) analogs. Exenatide clearance by the kidney is reduced in CKD and its use has been associated with acute kidney injury or acceleration of CKD progression [79,80]. It is therefore not recommended if the eGFR is <30 mL/min/1.73 m^2^ [70]. Experience is limited with liraglutide that is mostly metabolized outside the kidney. It is now recommended to avoid using it when eGFR is <50 mL/min/1.73 m^2^ until more data are available on its safety and risk of hypoglycemia [70,81]. Albiglutide and dulaglutide are new once weekly GLP-1 analogs. Experience of their use in patients with CKD is limited. In clinical pharmacology studies, there has been modest increase in their plasma concentration when used in type 2 diabetic patients with CKD [82,83]. There were also more hypoglycemia (when used in combination with insulin or insulin secretagogues) and more gastrointestinal side effects in this patient population [82,83,84]. The FDA has approved both drugs for patients with CKD without dose adjustment but the European Medicines Agency recommended avoiding their use in patients with GFR <30 mL/min/1.73 m^2^ and in patients on dialysis [83,84,85,86]. More data are expected in the future to clarify their safety further in patients with CKD.
- Sodium-glucose co-transporter 2 (SGLT2) inhibitors. Dapagliflozin, canagliflozin and empagliflozin do not increase the risk of hypoglycemia but are associated with increased risk of hypovolemic side effects in people with moderate to severe renal impairment, who are elderly (>70 years of age), and taking loop diuretics. Furthermore, because their efficacy decreases as renal function decreases, their use is restricted to patients with eGFR >45 (canagliflozin and empagliflozin) and >60 (dapagliflozin) mL/min/1.73 m^2^ [71,87,88,89,90].
- Insulin. There are no restrictions on the use of insulin in patients with renal disease, and clinically significant reductions in renal insulin metabolism are uncommon in patients with eGFRs >20 mL/min/1.73 m^2^ [50]. Nevertheless, people with severe renal disease (eGFRs <30 mL/min/1.73 m^2^) may have reduced glycogen stores and a reduced supply of gluconeogenic substrates, resulting in diminished capacity of the liver and kidney to release glucose and reverse insulin-mediated hypoglycemia. For all of the above considerations, insulin requirements may decrease by 20% or more when GFRs decrease below 45 mL/min/1.73 m^2^ [51]. Insulin requirements are often lower the day after hemodialysis [52]. The reduction varies among patients and regimen therefore must be individualized. Furthermore, special consideration needs to be made in people undergoing peritoneal dialysis depending on composition of the dialysate and mode of dialysis (continuous *versus* intermittent) [52].

## 8. Conclusions

Hypoglycemia is often the rate-limiting factor in achieving optimal glycemic control in patients with diabetes and is associated with substantial morbidity and mortality. 

CKD with a GFR <60 mL/min/1.73 m^2^ is found in up to 40% of people with diabetes. It is an independent risk factor for hypoglycemia, augments the risk for hypoglycemia that is already present in people with diabetes, and increases the risk of cardiovascular disease and death.

In addition to impaired hormonal counterregulation, people with CKD may have other risk factors for hypoglycemia, such as altered drug metabolism, albuminuria, malnutrition, impaired renal glucose release and insulin clearance, and dialysis associated problems.

Presence of CKD presents a challenge when deciding on appropriate antidiabetic drugs to use in patients with diabetes. Some agents (glipizide, meglitinides, DPP-4 inhibitors, thiazolidinediones, albiglutide, dulaglutide, orlistat, colesevelam, and insulin) can be used in all categories of CKD, provided they are used with caution or at a reduced dose. Other agents (metformin, glibenclamide (glyburide), glimepiride, gliclazide, exenatide, liraglutide, alpha glucosidase inhibitors, and SGLT2 inhibitors) are not recommended particularly in people with moderate to severe CKD (eGFR <45–60 mL/min/1.73 m^2^) because their efficacy is reduced and/or the risks of hypoglycemia or other adverse events are increased.

## Abbreviations

ACR: albumin-to-creatinine ratio
CKD: chronic kidney disease
DKD: diabetic kidney disease
eGFR: estimated glomerular filtration rate
KDIGO: Kidney Disease Improving Global Outcomes
T1DM: type 1 diabetes mellitus
T2DM: type 2 diabetes mellitus
